# Size and Topography of the Brain’s Functional Networks with Psychotic Experiences, Schizophrenia, and Bipolar Disorder

**DOI:** 10.1016/j.bpsgos.2024.100386

**Published:** 2024-09-07

**Authors:** Daniel Mamah, Shing Shiun Chen, Evan Gordon, Sridhar Kandala, Deanna M. Barch, Michael P. Harms

**Affiliations:** a Department of Psychiatry, Washington University Medical School, St Louis, Missouri; b Department of Radiology, Washington University Medical School, St Louis, Missouri; c Department of Psychological and Brain Sciences, Washington University Medical School, St Louis, Missouri

**Keywords:** Bipolar disorder, Functional connectivity, Human Connectome Project, Psychotic experiences, Schizophrenia

## Abstract

**Background:**

Existing functional connectivity studies of psychosis use population-averaged functional network maps, despite highly variable topographies of these networks across the brain surface. We aimed to define the functional network areas and topographies in the general population and the changes associated with psychotic experiences (PEs) and disorders.

**Methods:**

Maps of 8 functional networks were generated using an individual-specific template-matching procedure for each participant from the Human Connectome Project Young Adult cohort (*n* = 1003) and from a matched case cohort (schizophrenia [SCZ], *n* = 27; bipolar disorder, *n* = 35) scanned identically with the same Connectom scanner. In the Human Connectome Project Young Adult cohort, PEs were estimated based on scores from the Achenbach Self-Report Scale. The relationship of symptoms to the probability of network representation at each cortical vertex was assessed using logistic regression.

**Results:**

In Human Connectome Project Young Adult participants, PE severity on the Achenbach thought problems scale was predicted by increased language network (LAN) and dorsal attention network (DAN) areas and decreased cingulo-opercular network area (*r* < 0.12). Significant effects were found in SCZ, with a larger DAN and LAN and a smaller frontoparietal network. Network pattern analysis in SCZ showed an increased probability of LAN in the posterior region of the left superior temporal gyrus and of the visual network in the left insula. Regression analyses in SCZ found that mood dysregulation was related to increased DAN surface area.

**Conclusions:**

Those with PEs and SCZ showed abnormal functional network cortical topographies, particularly involving DAN and LAN. Network findings may predict psychosis progression and guide earlier intervention.

## Plain Language Summary

Studying the brain’s functional connectivity pattern can reveal how it becomes abnormal in psychiatric illness. Here, the authors describe a method of measuring this connectivity in those with schizophrenia and psychotic experiences, based on the size and topography of similarly connected brain regions (networks). The authors found that 2 networks were larger in psychosis, networks involved in attention control and language. In the future, these methods could be used to help predict psychosis progression and guide earlier intervention.

Psychotic experiences (PEs) exist on a severity continuum in the general population (1, 2, 3), with a prevalence of 5% to 30% found in most studies of largely adult populations (4, 5, 6, 7, 8), although substantially higher rates have been reported using more sensitive instruments (9). PEs increase the risk of developing psychotic disorders later in life (10, 11, 12, 13) and are often associated with anxiety, mood, and substance use disorders (14, 15, 16) and suicidal behavior (17,18).

Despite much progress in our understanding of its clinical correlates, the underlying pathophysiology of psychosis is still unclear (19,20). Identification of brain-based mechanisms underlying PEs has implications in the early identification of youths at high risk of developing psychotic disorders, potentially preventing the first episode of psychosis (21, 22, 23, 24). Among the most promising brain-based biomarkers has been resting-state functional magnetic resonance imaging (rsfMRI), which measures spontaneous low-frequency fluctuations in the blood oxygen level–dependent (BOLD) signal at rest to investigate the correlation of brain activity across different regions (25,26). Multiple studies have investigated resting-state functional connectivity in schizophrenia (SCZ) (27, 28, 29, 30, 31), and although results have been variable, they generally have involved diffuse hypoconnectivity across various brain regions indicating dysregulation of brain networks (27,30,31). There have also been a few rsfMRI studies in individuals with PEs or those at clinical high risk for developing psychosis, a criterion-based syndrome usually associated with attenuated PEs (21,32).

Methodological constraints can confound the results of functional connectivity studies. In most studies, parcellations of brain regions into functional networks are derived from group-averaged data (33, 34, 35, 36, 37). This is problematic given that there is substantial variation in functional network topography across individuals, although such functional networks are quite stable within individuals (38, 39, 40, 41, 42, 43, 44). Not accounting for each individual’s unique network topography means selected brain regions can be misaligned to assigned functional networks. This contributes to systematic noise and requires larger samples to attain the desired effect size (45).

Metrics used in rsfMRI studies typically involve investigations of connectivity strengths within or between functional network regions and do not include brain surface area and topographical patterns of brain systems. Given that functional connectivity using group-derived regions of interest is prone to errors due to variations in brain regions associated with specific functional networks across the population (38, 39, 40, 41, 42, 43, 44, 45), analyses of functional network topography could be more interpretable and have better clinical utility (46). Identification of the unique functional network topography of an individual’s brain could have implications for personalized medicine (45) and has already shown clinical utility in surgical planning (47) and brain stimulation therapies across diverse psychiatric conditions (26,48). Abnormal patterns of functional networks, if present early in the course of the disorder and in those with PEs, could also have implications in early identification and prevention. In addition, network size could provide insights into the extent to which specific brain networks are being used. A relatively small functional network in a participant indicates the presence of fewer brain regions with an underlying connectivity pattern characteristic of that network. This may be related to a deprioritization of the associated brain function (e.g., voluntary control of visuospatial attention for the dorsal attention network [DAN], executive functioning for the frontoparietal network [FPN]). Conversely, larger networks may indicate a prioritization of its associated functions in the brain.

Studies have found that precise mapping of an individual’s unique functional brain network requires at least 40 to 60 minutes of resting-state data (39,41); however, that much data are very rarely collected in research due to cost and participant burden. Others have noted that, in some cases, shorter-duration resting scans of approximately 20 minutes can be useful for determining individual-specific brain networks with only slightly less precision than longer scans (22,23); however, the reduced relative influence of temporal autocorrelation in the time series data with longer duration acquisitions can improve reliability (41). Among the existing large datasets, only the Human Connectome Project Young Adult (HCP-YA) acquired data of this long duration, specifically, four 15-minute rsfMRI runs to provide more stable connectivity estimates (49). HCP-YA participants were scanned using a customized Siemens 3T Skyra (Connectom scanner) with a 32-channel head coil at Washington University in St. Louis (50). Therefore, the HCP-YA dataset can be a unique resource for identifying associations of functional network connectivity metrics with behavioral traits, including PEs. Using the same Connectom scanner and identical acquisition protocol as the HCP-YA, our group also acquired 60 minutes of BOLD imaging data from a cohort of young adults with SCZ or bipolar disorder (BPD). Therefore, these datasets provide an opportunity to investigate unique features of brain connectivity in psychiatric disorders, which are directly comparable with those obtained through the HCP-YA.

In this study, we investigated the functional network topography of 1003 HCP-YA healthy participants, using measures of cortical surface area and distribution of the 8 largest and most widely studied functional networks: the default mode network (DMN), FPN, visual network (VIS), DAN, language network (LAN), cingulo-opercular network (COP), somatosensory-body network, and somatosensory-face network. We then studied the relationship of PEs with functional topography metrics in this HCP-YA cohort. Finally, we studied these metrics in a cohort of participants with SCZ and BPD who were scanned identically as the HCP-YA cohort using the Connectom scanner. Results from our study may lead to a greater understanding of functional brain organization with PEs and psychotic disorders.

## Methods and Materials

### Participants

Two participant groups were investigated: 1) a HCP-YA cohort and 2) a Connectom-scanned clinical cohort of young adults with SCZ or bipolar I disorder (BPD), both psychotic and nonpsychotic.

The HCP-YA cohort consisted of 1003 participants (ages 22–37 years) from whom high-quality neuroimaging data were collected using a customized Connectom scanner at Washington University (50). Potential participants were excluded if they or their siblings had a severe neurodevelopment disorder, neurological disorder, or other physical disorder that could influence brain function (50,51).

The clinical cohort consisted of 27 participants with SCZ and 35 participants with BPD, 18 to 35 years of age, imaged using the same Connectom MRI scanner at Washington University as the HCP-YA cohort, and with an identical scanning protocol. They were diagnosed based on a master’s level clinical research coordinator who used the Structured Clinical Interview for DSM-IV Axis I disorders (52). All participants were excluded if they 1) met DSM-IV criteria for substance dependence or severe/moderate abuse during the previous 3 months, 2) had a clinically unstable or severe general medical disorder, or 3) had a history of head injury with documented neurological sequelae or loss of consciousness.

Written informed consent was obtained before participation, and all study protocols were approved by the Institutional Review Board at the Washington University School of Medicine in St. Louis.

### Behavioral Assessments

For HCP-YA participants, 2 syndrome scales from the 126-item Achenbach Adult Self-Report Scale (53) were used: the thought problems (TP) scale and the anxious/depressed scale. The TP scale (54) consists of 10 items common in several mental disorders, including hallucinations, obsessions/compulsions, strange thoughts and behaviors, self-harm, and suicide attempts (see Box 1), and the anxious/depressed scale consists of 18 items. Each item has 3 response categories (0 = not true, 1 = somewhat or sometimes true, or 2 = very true or often true) based on the preceding 6 months. To examine relationships with specific PEs, specific questions from the TP scale were identified, as previously described (55): 1) “I hear sounds or voices that other people think aren’t there,” Q.40; 2) “I see things that other people think aren’t there,” Q.70; 3) “I do things that other people think are strange,” Q.84; and 4) “I have thoughts that other people would think are strange,” Q.85. A total of 928 HCP-YA participants completed the 126-item Achenbach Adult Self-Report Scale. Of these, 689 (74.2%) endorsed symptoms on the TP scale, and 216 (23.3%) endorsed at least 1 of 4 PEs. The number of participants endorsing specific PEs was 15 for Q.40, 13 for Q.70, 168 for Q.84, and 146 for Q.85.

Box 1 **Comprising Question Items on the Thought Problems Scale of the Achenbach Adult Self-Report Scale**

Achenbach Items: Thought Problems Scale

Q.9. “I can’t get my mind off certain thoughts”
Q.18. “I deliberately try to hurt or kill myself”
Q.36. “I accidentally get hurt a lot”
Q.40. “I hear sounds or voices that other people think aren’t there”
Q.46. “Parts of my body twitch/make nervous movements”
Q.63. “I would rather be with older people than people my own age”
Q.66. “I repeat certain acts over and over”
Q.70. “I see things other people think aren’t there”
Q.84. “I do things that other people think are strange”
Q.85. “I have thoughts that other people would think are strange”

Among SCZ and BPD participants, recent psychosis-related symptoms (i.e., in the previous 2 weeks) were assessed using the Scale for the Assessment of Negative Symptoms and the Scale for the Assessment of Positive Symptoms (56). Long-term symptoms (i.e., in the previous 12 months) were assessed using the 16-item Washington Early Recognition Center Affectivity and Psychosis Screen, a self-report questionnaire that assesses psychosis and affective symptom severity based on symptom frequency and functioning (9,57, 58, 59).

### Imaging Procedure

The HCP-YA participants and participants with SCZ and BPD were scanned identically (60,61). Briefly, participants were run on a customized Siemens Connectom 3T scanner with a 32-channel head coil and completed T1-weighted and T2-weighted structural scans (0.7 mm isotropic) and four 15-minute resting-state BOLD scans, with their eyes open and fixated on a crosshair. Resting-state scans were acquired using a T2∗-weighted multiband (8) echo-planar imaging sequence with 72 axial slices per volume, 2 × 2 × 2-mm voxels, field of view = 208 mm, echo time = 33.1 ms, repetition time = 720 ms, and flip angle = 52°.

### Preprocessing Pipelines

fMRI data from all participants were run through minimal preprocessing pipelines (60). Subsequently, CIFTI-format resting-state data (gray matter surface vertices plus subcortical gray matter voxels, collectively termed grayordinates) underwent additional preprocessing. Six rigid body motion parameters, their first derivatives, and their squares were used as regressors to correct for motion, and additional denoising was performed using FMRIB’s Independent Component Analysis–based X-noiseifier (62,63). For removing the artifact components, first, we aggressively regressed out the 24 motion-estimation (6 rigid body motion parameters, their temporal derivatives, and the squares of the 12 resulting regressors) confound time series (*Cm*) from the data (*cts*) and independent component analysis (*ICA*) component time series:

(1) $$\begin{array}{c} \{cts=Cm\cdot \left(pinv\left(Cm\right)\cdot cts\right)cts=Cm\cdot pinvCm\cdot cts\text{,}\\ ICA=Cm\cdot \left(pinv\left(Cm\right)\cdot ICA\right)ICA=Cm\cdot pinvCm\cdot ICA\} \end{array}$$

Then, the regression of the motion-cleaned ICA component time series was multiplied by the motion-cleaned data to design the unique matrix for denoising:

(2) $$\left\{\beta ICA=pinv\left(ICA\right)\cdot cts\beta \text{ICA}=\text{pinvICA·cts}\right\}$$

Finally, the designed matrix was used to remove the bad components from the data:

(3) $$\{cts=cts\;-\;\left(ICA\left(bad\right)\cdot \beta ICA\left(bad\right)\right)\text{cts}=\text{cts}\;-\;\text{ICAbad·}\beta \text{ICAbad}\}$$

Mean grayordinate time course was then regressed from the time series of each grayordinate, followed by temporal bandpass filtering (0.009–0.08 Hz). All 4 resting-state runs were concatenated following preprocessing, yielding 60 minutes (4800 frames) of resting-state data.

### Functional Network Template Matching

The template-matching procedure has been previously described (64) and depicted in Figure S1. Briefly, connectivity templates were built for each network in a previously described group-average network map (41,64). In each participant, we identified that participant’s versions of group-average networks by matching each vertex’s participant-specific connectivity pattern to the set of templates described above. We first correlated the vertex’s time course with all other gray matter time courses in that participant’s data, Fisher transformed the resulting connectivity map, and then thresholded and binarized the resulting map at the top 5% of connectivity values (across vertices). This resulted in a binarized map of regions with high connectivity to the chosen cortical vertex.

**Figure 1:**
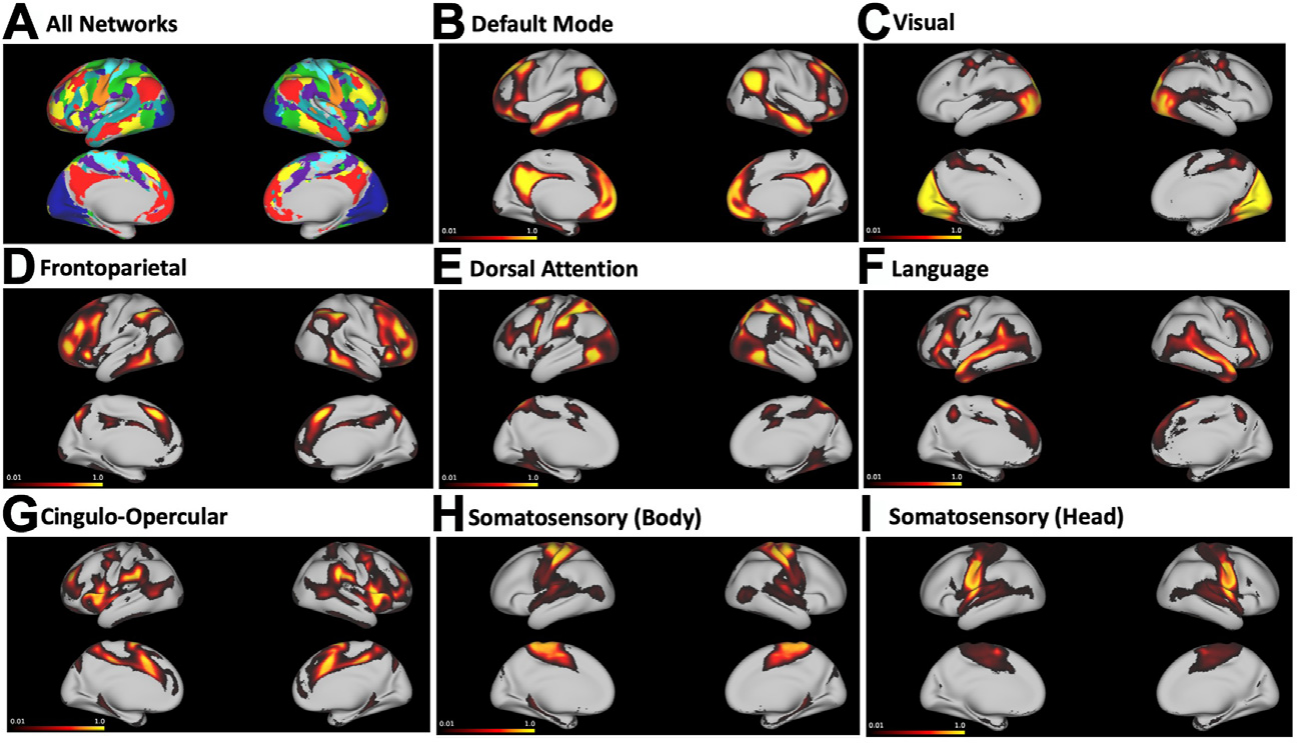
Variability in functional network topography across healthy individuals. **(A)** Colorized brain maps of all 8 functional networks derived from individual Human Connectome Project Young Adult participants, with 6 mm smoothing applied. Networks include default mode (red), visual (dark blue), frontoparietal (yellow), dorsal attention (green), language (cyan), cingulo-opercular (purple), somatosensory body (light blue), and somatosensory face (orange). **(B–H)** Probability maps of the cortical surface distribution of the 8 functional networks across the Human Connectome Project Young Adult participants (*n* = 1003). Bright yellow indicates regions where the network is located in close to 100% of participants. Black/dark red regions indicate a lower probability of network localization. Regions where a network is localized in <1% of participants are not included in the probability maps.

The resulting data were spatially smoothed at full width at half maximum (6 mm) (Figure 1A).

### Statistical Analysis

An a priori power analysis with 2 groups was conducted in G∗Power (65) to establish the sample size required for an alpha of 0.05, a power of 0.8, and a medium effect size (*d* = 0.6), due to effects seen in some psychosis functional connectivity studies (66,67), with an allocation ratio of 37. Based on these assumptions, the desired sample sizes were 22 and 832, respectively. All other statistical analyses were done using SAS 9.4 (SAS Institute Inc.).

Group comparisons of functional network surface area (after smoothing) were done using analysis of variance (ANOVA), with and without covarying for age, sex, and mean BOLD image signal-to-noise ratio. Bonferroni corrections were applied to control for type I errors, accounting for the 8 functional networks analyzed. A corrected *p* value < .00625 was used for statistical significance (.05/8). Group comparisons of functional network topography were done using χ^2^ tests at each brain voxel, comparing the rate of the network localizing with that voxel between groups. To control the probability of finding any false positives and threshold the group comparison maps, previously described cluster correction was implemented (68). An empirical null distribution of the test statistic was generated by randomly permuting labels of participant connectivity maps 1000 times and computing *p* value from the χ^2^ statistic. The corrected *p* values are identified by the location where the *p* < .05 and the cluster size is larger than the cluster extent threshold.

Stepwise multiple regression, with forward selection, was used to investigate potential network size predictors of clinical scores and estimate the relationship of functional network surface area with clinical scores. The 8 functional network areas were dependent variables in the model. Considering data overfitting and inflated type I error that can occur with this method, results were considered exploratory. In addition, Pearson’s correlations were conducted to investigate these relationships. To investigate the relationship between functional network topography and clinical scores, logistic regressions were done at each brain voxel, analyzing the odds of a specific network localizing to that voxel with unit increases in scores.

## Results

### Demographic and Clinical Profiles

The profiles of the groups scanned using the Connectom scanner are presented in Table 1. HCP-YA participants were slightly older (mean, 28.7 years) than participants with SCZ and BPD (mean, ∼25 years). The ratio of males to females was higher in SCZ than in the other groups. Black was the predominant ethnicity in SCZ, whereas the other groups’ most predominant ethnicity was White.

**Table 1:** Demographic and Brain Image Characteristics of HCP-YA and Clinical Cohorts

Characteristic	HCP-YA, *n* = 1003	Bipolar Disorder, *n* = 35	Schizophrenia, *n* = 27	*F*/χ^2^	*p*
Age, Years	28.7 (3.7)	26.3 (3.0)	25.1 (3.3)	19.3	<.0001
Sex					
Female	530 (53.2%)	18 (51.4%)	6 (22.2%)	10.1	.006
Male	470 (46.8%)	17 (48.6%)	21 (77.8%)		
Ethnicity					
Asian	63 (6.3%)	3 (8.6%)	1 (3.7%)	52.1	<.0001
Black	139 (13.9%)	5 (14.3%)	17 (63.0%)		
Mixed	24 (2.4%)	1 (2.9%)	1 (3.7%)		
White	758 (75.6%)	25 (71.4%)	7 (26.0%)		
Other^a^	19 (1.9%)	1 (2.9%)	1 (3.7%)		
Education, Years	15.0 (1.8)	15.4 (2.7)	12.9 (1.5)	18.0	<.0001
Total Gray Volume, cm^3^	706 (67)	707 (63)	702 (53)	0.1	.99
Cortical Gray Volume, cm^3^	525 (54)	524 (50)	518 (424)	0.2	.9
Total White Volume, cm^3^	443 (56)	441 (59)	447 (53)	1.2	.3
Magnetic Resonance Image Noise-to-Signal Ratio	4.43 (1.6)	4.78 (2.0)	5.67 (3.7)	7.6	.0006

Values are presented as mean (SD) or *n* (%). Analyses were done using either 3-way analysis of variance or χ^2^ tests.

a Includes other ethnicities and non-responses.

Table S1 compares the profiles of HCP-YA participants with and without a history of PEs.

### Surface Area and Topography of Functional Networks in HCP-YA

The mean (SD) relative cortical surface size of the 8 functional networks in the HCP-YA participants, expressed as percentages of the total cortical area in decimals, were as follows: DMN, 0.183 (0.03); VIS, 0.161 (0.02); FPN, 0.123 (0.02); DAN, 0.099 (0.02); LAN, 0.073 (0.02); COP, 0.082 (0.02); somatosensory-body, 0.075 (0.02); and somatosensory-face, 0.045 (0.02).

The probability of cortical vertex representation for each network across the entire HCP-YA cohort (*n* = 1003) is shown in Figure 1B–I.

### Psychotic-Like Experiences and Functional Network Size and Topography in HCP-YA

Using stepwise regression, there were no functional networks that predicted symptom severity on the anxious/depressed scale. On the TP scale, only increased LAN area (partial *R*^2^ = 0.0125, *r* = 0.112, *F*_1,939_ = 11.87, *p* = .0006) and increased DAN area (partial *R*^2^ = 0.007, *r* = 0.084, *F*_1,939_ = 6.78, *p* = .0093) predicted symptom severity. The result of Pearson’s correlation of network areas with the 2 Achenbach scales is presented in Table 2. The table also shows significant inverse correlations of the TP scale symptoms with COP area (*r* = −0.088, *p* = .007).

**Table 2:** Pearson’s Correlation Results of Clinical Symptom Scores With Functional Network Size

Network	ASR Depression/Anxiety	ASR Thought Problems
Default Mode		
*r*	−0.005	−0.048
*p*	.87	.14
Visual		
*r*	−0.053	−0.063
*p*	.10	.054
Frontoparietal		
*r*	0.020	0.018
*p*	.54	.58
Dorsal Attention		
*r*	0.049	0.095
*p*	.13	.0035^a^
Language		
*r*	0.046	0.112
*p*	.16	.0006^a^
Cingulo-Opercular		
*r*	−0.041	−0.088
*p*	.21	.007^b^
Sensorimotor Body		
*r*	−0.054	−0.010
*p*	.098	.76
Sensorimotor Face		
*r*	−0.017	0.025
*p*	.60	.45

ASR, Achenbach Adult Self-Report.

a *p* < .005.

b *p* < .05.

Post hoc analyses investigated relationships of DAN and LAN area with 4 isolated psychotic-like experience (PLE) symptoms within the TP scale (55), as shown in Table 3. Larger LAN correlated with auditory hallucinations (*r* = 0.115, *p* = .0004), whereas smaller COP area was related to strange actions (*r* = −0.089, *p* = .006) and thoughts (*r* = −0.093, *p* = .004). There were no significant relationships of PLEs with DAN area. Pearson’s correlation results between all 8 functional network areas and PLEs are summarized in Table S2.

**Table 3:** Pearson’s Correlations of Psychotic-Like Experiences From the Achenbach Adult Self-Report With DAN and LAN Size in HCP-YA Participants (*n* = 1003)

Achenbach Items	DAN		LAN		Cingulo-Opercular Network	
	*r*	*p*	*r*	*p*	*r*	*p*
Q.40. “I hear sounds or voices that other people think aren’t there”	0.003	.9	0.115	.0004^a^	−0.079	.015^b^
Q.70. “I see things other people think aren’t there”	0.016	.6	0.082	.012^b^	−0.062	.058
Q.84. “I do things that other people think are strange”	0.052	.11	0.066	.045^b^	−0.089	.006^b^
Q.85. “I have thoughts that other people would think are strange”	0.020	.55	0.02	.55	−0.093	.004^c^

DAN, dorsal attention network; HCP-YA, Human Connectome Project Young Adult; LAN, language network.

a *p* < .0005.

b *p* < .05.

c *p* < .005.

**Figure 2:**
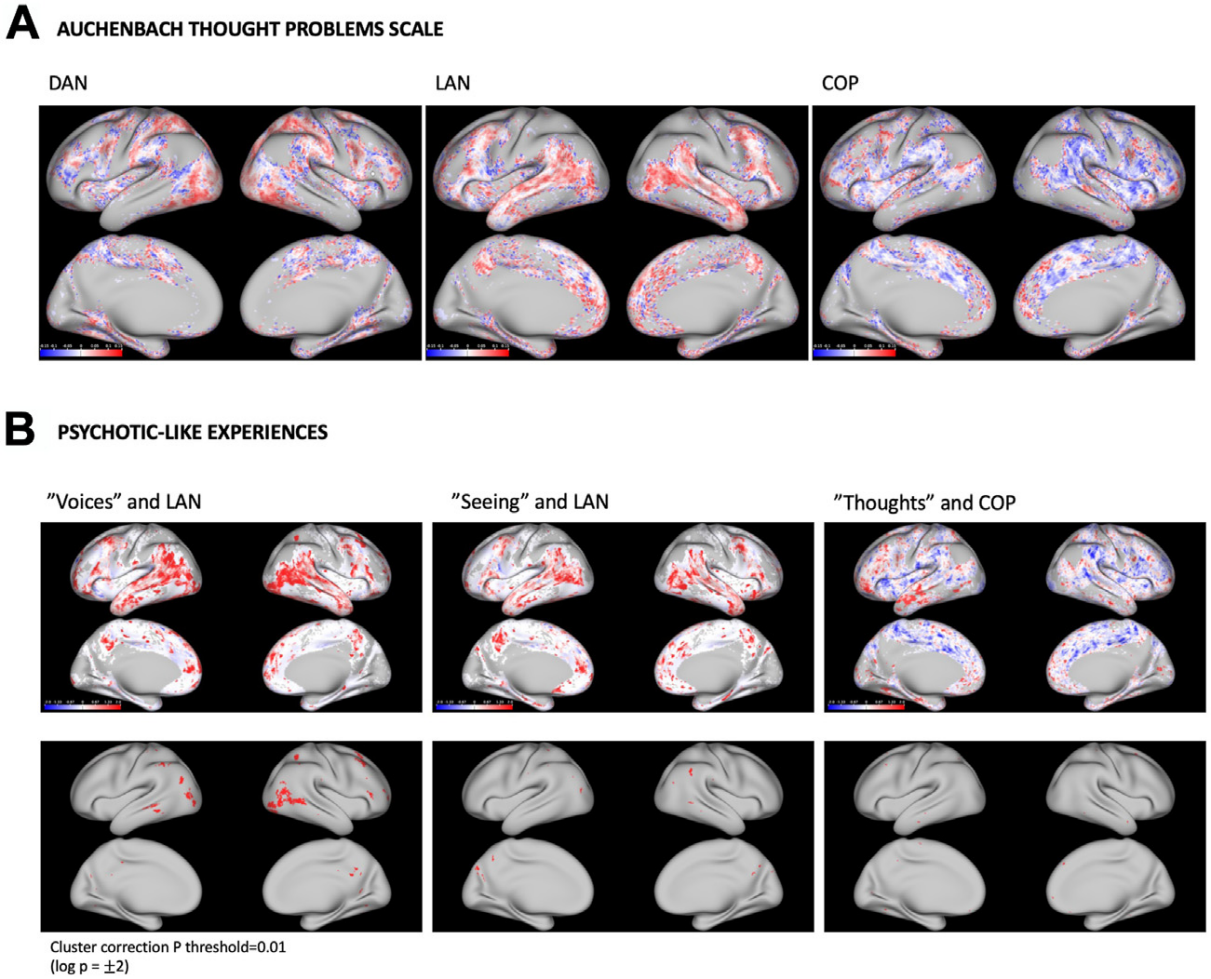
Relationships of psychotic-like experiences (PLEs) to functional network topography. **(A)** Results of logistic regressions at each brain voxel, analyzing the odds of a functional network localizing to that voxel with unit increases in scores on the Achenbach thought problems scale among Human Connectome Project Young Adult participants. Odds ratios have been log-transformed. Increased odds of a network localizing to a brain voxel with increased symptoms are shown in red. Decreased odds of a network localizing to a brain voxel with increased symptoms are shown in blue. **(B)** The results of χ^2^ tests at each brain voxel, comparing the rate of the network localizing with that voxel, in those with and those without PLEs. “Hearing” pertains to hearing sounds or voices others do not (question 40). “Seeing” pertains to seeing things others do not (question 70). “Thoughts” pertains to experiencing strange thoughts (question 85). Red indicates voxels where the network localizes more frequently in those with PLEs (compared with those without). Blue indicates voxels where the network localizes less frequently in those with PLEs (compared with those without). Cluster-based correction has been applied to the bottom figures (uncorrected threshold, *p* < .001). COP, cingulo-opercular network; DAN, dorsal attention network; LAN, language network.

The association of DAN, LAN, and COP topography with increased TP scale symptoms is shown in Figure 2A. In particular, this involved logistic regressions at each brain voxel to analyze the odds of the network localizing to that voxel with unit increases in TP scores. Figure 2B compares LAN and COP topography in those with PLEs with those without these experiences, estimating the frequency of network representation at each voxel using χ^2^ tests.

### Functional Network Surface Area in SCZ and BPD

**Figure 3:**
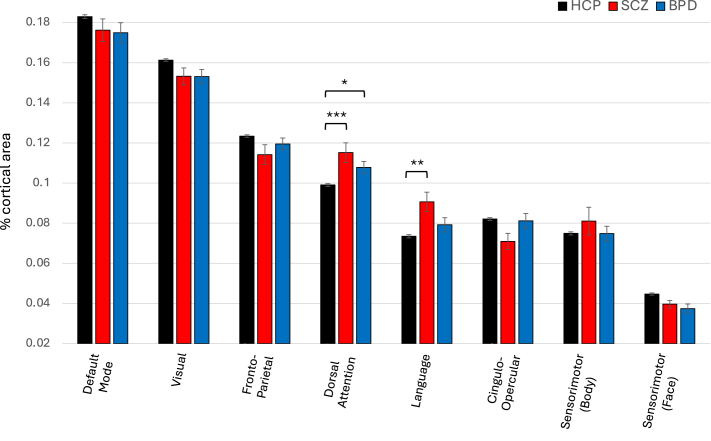
Size of functional networks in schizophrenia (SCZ) and bipolar disorder (BPD). **(A)** Comparison of the average cortical surface areas of 8 functional networks (relative to the total cortical area) across participants with SCZ (*n* = 27) and BPD (*n* = 35) and healthy control participants from the Human Connectome Project (HCP) Young Adult cohort (*n* = 1003). ∗*p* < .05, ∗∗*p* < .005, ∗∗∗*p* < .0005.

Group means of functional network cortical surface areas are presented in Table 4 and Figure 3. A statistically significant (*p* < .00625) omnibus effect was observed for DAN (*F*_2,5_ = 10.60, *p* < .0001) and LAN (*F*_2,5_ = 7.20, *p* = .0008). Post hoc ANOVA found significant effects involving SCZ versus HCP-YA comparisons, with larger DAN (*F*_1,6_ = 15.6, *p* < .0001) and LAN (*F*_1,6_ = 12.7, *p* = .0004) in SCZ and larger DAN in BPD (*F*_1,6_ = 6.1, *p* = .014).

**Table 4:** Group Differences in Functional Network Brain Surface Area Across HCP-YA, BPD, and SCZ Populations

Network	HCP-YA, *n* = 1003	BPD, *n* = 35	SCZ, *n* = 27	*F*^a^	*p*
Default Mode	0.183 (0.03)	0.175 (0.03)	0.176 (0.03)	0.9	.4
Visual	0.161 (0.02)	0.153 (0.02)	0.153 (0.02)	3.2	.02
Frontoparietal	0.123 (0.02)	0.120 (0.02)	0.114 (0.03)	3.6	.01
Dorsal Attention	0.099 (0.02)	0.108 (0.02)^b^	0.115 (0.03)^b^	5.8^c^	.0007^c^
Language	0.073 (0.02)	0.079 (0.02)	0.091 (0.03)^b^	2.7^c^	.047^c^
Cingulo-Opercular	0.082 (0.02)	0.081 (0.02)	0.071 (0.02)	0.7	.5
Sensorimotor Body	0.075 (0.02)	0.075 (0.02)	0.081 (0.04)	0.4	.8
Sensorimotor Face	0.045 (0.02)	0.0375 (0.01)	0.040 (0.01)	1.7	.2

Values are presented as mean (SD), expressed as percentages of the total cortical area in decimals.

BPD, bipolar disorder; HCP-YA, Human Connectome Project Young Adult; SCZ, schizophrenia.

a Type III sum of squares used in analysis of variance.

b Groups showing significant results against HCP-YA.

c Statistically significant (*p* < .0063) results.

Including age, sex, and signal-to-noise ratio as additional covariates showed a highly similar result, with a significant overall group effect for DAN (*F*_2,3_ = 10.9, *p* < .0001) and LAN (*F*_2,3_ = 7.4, *p* = .0006) (see Figure S2). Post hoc ANOVA found significant effects involving SCZ versus HCP-YA comparisons, with larger DAN (*F*_1,4_ = 16.0, *p* < .0001) and LAN (*F*_1,4_ = 13.1, *p* = .0003) in SCZ and larger DAN (*F*_1,4_ = 5.2, *p* = .02) in BPD.

Results of group analyses, involving only those HCP-YA participants without a history of PEs (*n* = 712), are presented in Table S3. A statistically significant (*p* < .00625) omnibus effect was observed for VIS (*F*_2,5_ = 5.1, *p* = .006), DAN (*F*_2,5_ = 11.0, *p* < .0001), and LAN (*F*_2,5_ = 8.2, *p* = .0003). Post hoc ANOVA found significant effects involving SCZ versus HCP-YA comparisons, with smaller VIS (*F*_1,6_ = 4.6, *p* = .03) and larger DAN (*F*_1,6_ = 16.1, *p* < .0001) and LAN (*F*_1,6_ = 14.4, *p* = .0002) in SCZ and smaller VIS (*F*_1,6_ = 6.0, *p* = .01) and larger DAN (*F*_1,6_ = 6.6, *p* = .01) in BPD.

### Functional Network Topography in SCZ and BPD

The probability of cortical vertex representation for each network across the SCZ and BPD is shown in Figure S3.

**Figure 4:**
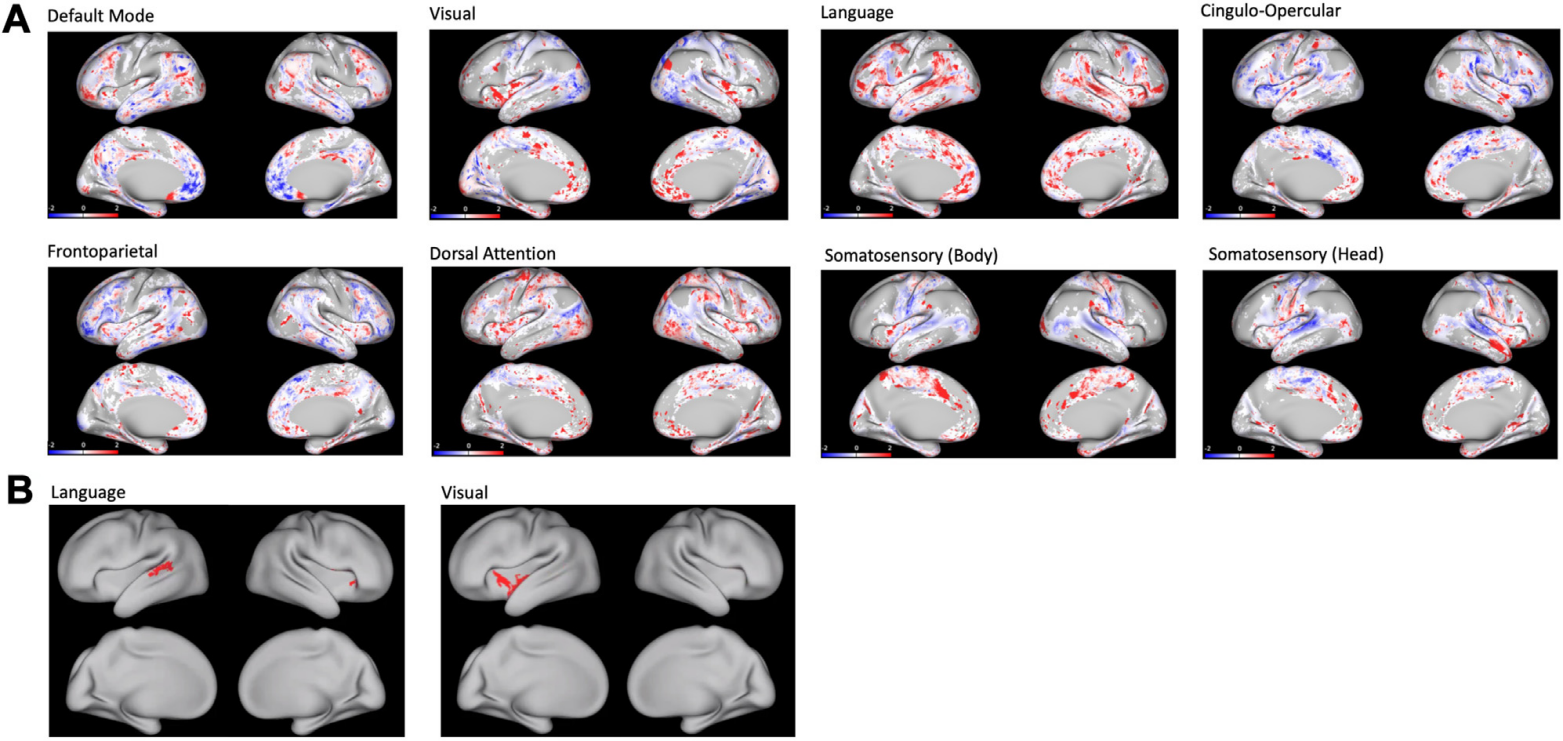
Functional network topography in schizophrenia. **(A)** The figures are the results of χ^2^ tests at each brain voxel, comparing the rate of the network localizing to that voxel in participants with schizophrenia (*n* = 27) with that voxel in Human Connectome Project Young Adult (healthy control) participants (*n* = 1003). Color coding is based on log*p* values. Red shading indicates voxels where the network localizes more frequently in participants with schizophrenia. Blue shading indicates voxels where the network localizes more frequently in Human Connectome Project Young Adult participants. **(B)** Significant results after cluster-based correction in the language and visual networks (cluster-based correction results for other networks can be found in the Supplement).

Network surface pattern differences in HCP-YA versus SCZ based on the likelihood of cortical vertex representation are shown in Figure 4A. After cluster-wise correction, statistical significance was most notable in a posterior region of the left superior temporal cortex in LAN and the left insula of VIS, as shown in Figure 4B. Cluster-wise corrected results for all networks are shown in Figure S4.

HCP-YA versus BPD comparisons are shown in Figure S5, with no region surviving cluster-wise correction.

### Clinical Relationships With Functional Network Topography in Participants With SCZ

**Figure 5:**
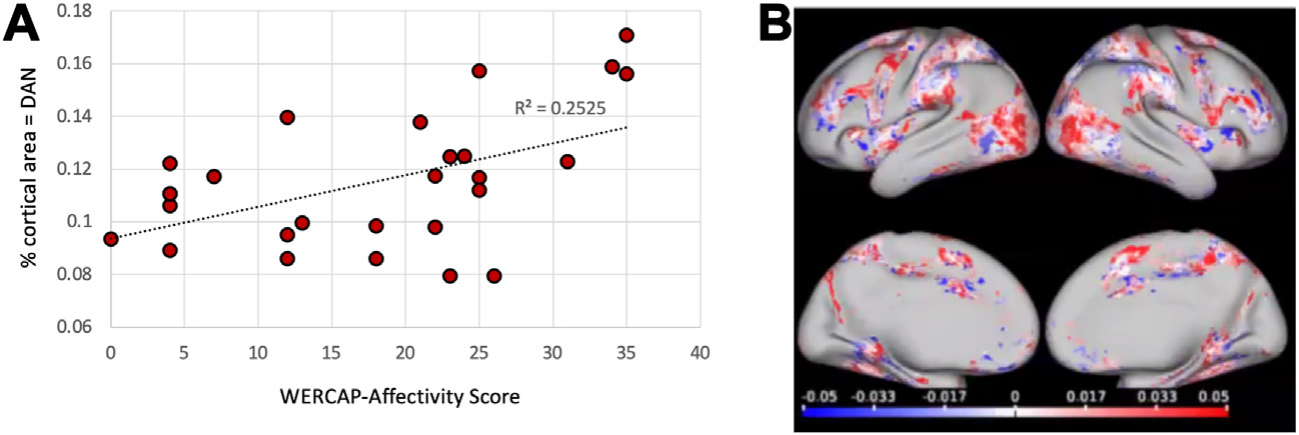
Correlations of mood symptoms with dorsal attention network (DAN) size and topography. **(A)** Graph depicting the relationship between the relative size of DAN and scores on the affectivity section on the Washington Early Recognition Center Affectivity and Psychosis Screen (WERCAP-affectivity) in participants with schizophrenia (*n* = 27). WERCAP-affectivity measures the severity of mood dysregulation. **(B)** Results of logistic regressions at each brain voxel, analyzing the odds of DAN localizing to that voxel with unit increases in scores on the WERCAP-affectivity in participants with schizophrenia. Odds ratios have been log-transformed. Increased odds of DAN localizing to a brain voxel with increased mood symptoms are shown in red. Decreased odds of DAN localizing to a brain voxel with increased mood symptoms are shown in blue.

Using stepwise regression, increased Washington Early Recognition Center Affectivity and Psychosis–affectivity scores predicted increased DAN area (*R*^2^ = 0.253, *r* = 0.5, *F*_1,26_ = 6.71, *p* = .0076). The clinical relationship is depicted in Figure 5A and with network patterns in Figure 5B. Pearson’s correlation results between all functional network areas and clinical scores are summarized in Table S4.

## Discussion

Our study investigated the cortical functional network topography of a large youth population derived from the HCP, using a template-matching process. We characterized functional networks firstly by measuring their surface areas. While this does not directly measure connectivity strength within or between networks, it seems to provide information on how different brain functions are prioritized. We found that, in healthy participants, DMN was the largest among the functional networks, occupying approximately 18% of the cerebral cortex. The role of DMN is not entirely clear. Most active at rest, DMN is believed to be involved in internally focused thought processes such as daydreaming, self-reflection, and envisioning the future (69,70).

In the healthy HCP-YA cohort, we found a relationship between functional network topography of LAN and COP and PLEs. LAN surface area correlated mostly with auditory hallucinations, whereas the strongest relationship of COP surface area was with strange thoughts and strange actions. Analysis of the network topography showed that the superior temporal gyrus region of LAN was most strongly related to hallucinations. Encompassing Broca’s and Wernicke’s areas and superior temporal cortical regions, LAN serves a complex function related to auditory perception, language comprehension, and speech production (71). Although speculative, a large superior temporal LAN with PEs as observed in our study may indicate erroneous overprocessing of tasks, resulting in auditory or visual misperceptions. The observed relationship of smaller COP surface area with strange thoughts and strange actions suggests a role of this network in delusional thinking and disorganized behaviors, respectively. COP is thought to be involved in the stable maintenance of task control and goals (72,73). However, others have proposed that visceral, autonomic, and sensory data are integrated by COP to assess the homeostatic relevance or salience of internal and external stimuli (74). This would be consistent with our findings, suggesting that COP is required for logical thought processing and abnormalities of this network would result in misperceptions or delusional thoughts. In line with this, hypoconnectivity of COP has been reported in SCZ (75), and a significant negative association with COP global efficiency has been found with PEs in a subgroup of HCP-YA participants (55). Interestingly, the DAN surface area did not correlate with specific PEs, suggesting that its observed relationship with thought problems in our studies may have been driven by the nonpsychotic components of the TP scale, including intrusive thoughts, impaired peer relationships, and repetitive behaviors.

We analyzed hour-long resting-state functional data from patients with SCZ and BPD, the longest dataset from these populations to our knowledge. In the SCZ group, both LAN and DAN areas were larger than in the control group while FPN was smaller. These findings were similar to those observed with PLEs in the HCP-YA population. Notably, the larger LAN in SCZ was most pronounced in the left superior temporal cortical region, suggesting an overrepresentation of functions attributable to LAN, including language processing and auditory perception, which may underlie characteristic auditory hallucinations. The superior temporal cortex is one of the brain regions most often found to show abnormalities in SCZ, including cortical thinning (76, 77, 78) and abnormal language task activation and hallucinations (78, 79, 80). Our study indicates that the topographical representation of auditory and language processing regions, i.e., LAN, can also be altered in SCZ. Interestingly, the size of LAN showed a slight inverse correlation with psychotic symptoms in patients with SCZ in our study, which suggests a more complex relationship of LAN size with symptoms, possibly one confounded by antipsychotic effects. Therefore, larger studies would be required to replicate findings and explore possible confounding variables.

We also found that the DAN area was also relatively enlarged in the SCZ group, similar to the association with TPs in the healthy HCP-YA population, suggesting this network may also have a role in regulating normal thought processing and perception. DAN, comprising regions that include the intraparietal sulcus and frontal eye fields, has been associated with orienting and maintaining attention toward relevant external information including that processed by VIS and exerts top-down attentional control during cognitive tasks (81,82). Deficits of attention control are a core feature of SCZ (83), and DAN has been reported to be overactive with a simple target detection task (84) and increased attentional demands (85), or to have distinct activity during visual perceptual organization (86), in affected patients. The centrality of DAN in healthy individuals has also been shown to increase during the recovery phase after acute stress exposure (87), and aberrant DAN connectivity has been related to symptom severity with posttraumatic stress disorder (88,89). Therefore, increased DAN surface area might be related to the amplification of attentional control in SCZ together with higher stress sensitivity, which is associated with psychoses (90,91). Interestingly, we also found that the most significant clinical relationship was observed between increased DAN cortical area and chronic mood symptoms.

There are several limitations to our study. Although the stability of resting-state functional network cortical “fingerprints” has been asserted (92), the reliability of cortical network size is unclear and was not explored in this study. In addition, there is likely some variation of network topography with different task states, based on the reported subtle task-related modification of functional network connectivity (93, 94, 95). Thus, determining the reliability of topographical measures under different conditions would be needed in future studies. Second, we found that estimating PEs in the HCP-YA using the Achenbach resulted in a much lower prevalence in the population than expected (6,9). Studies using questionnaires specifically designed for PEs such as the Prodromal Questionnaire (96) or the Washington Early Recognition Center Affectivity and Psychosis Screen (9,57,58) would likely identify more intricate relationships with brain network topography. However, such assessments were not conducted in the HCP-YA population and thus not available for this study. Third, the sample size of our patients with SCZ and BPD was relatively small, which limited the power for identifying small group effects. The relationship of potential confounders, such as medication or substance use, to our findings were also not explored due to the limited sample size of our case-control cohort, warranting studies involving larger SCZ datasets in the future.

### Conclusions

Our study found that the surface areas of specific cortical functional networks at rest differ in those with PEs from those without. These findings, particularly that of increased DAN and LAN, were also seen in individuals with SCZ, which may reflect abnormal connectivity of comprising brain regions. Topographical network measures may aid in the identification of those at risk of developing psychotic disorders. Large-scale studies are needed to identify the stability of functional brain network topography and confounding factors.

## Supplementary Material

Supplementary Data

Key Resources Table
