# Supplementary material for: Size and Topography of the Brain’s Functional Networks with Psychotic Experiences, Schizophrenia, and Bipolar Disorder
Source: Biol Psychiatry Glob Open Sci. 2024 Sep 7;4(6):100386. doi: 10.1016/j.bpsgos.2024.100386 (PMC11740805; doi:10.1016/j.bpsgos.2024.100386)
Supplement: Supplementary Data [file mmc1.pdf]

SUPPLEMENTARY INFORMATION

Size and Topography of the Brain's Functional Networks With Psychotic Experiences, Schizophrenia and Bipolar Disorder

Mamah *et al.*

Supplementary Table 1. Demographic and brain image characteristics of HCP-YA participants with and without psychotic experiences

Characteristic	Psychotic Experiences (n=216)	No Psychotic Experiences (n=712)	t/χ^2	p
**Age (s.d.)**	27.8 (3.7)	29.0 (3.7)	4.1	<0.0001
**Sex (%)**				
Female	82 (38.1)	405 (57.3)	24.2	<0.0001
Male	133 (61.9)	302 (42.7)		
**Ethnicity (%)**			24.3	<0.0001
Asian	17 (7.9)	41 (5.8)		
Black	39 (18.1)	85 (11.9)		
White	141 (65.3)	564 (79.2)		
Mixed race	13 (6.0)	11 (1.5)		
Other	6 (2.8)	11 (1.5)		
**Education, years (s.d.)**	14.7 (1.8)	15.1 (1.7)	1.1	0.003
**MR Image Noise-to-Signal Ratio**	4.45 (1.8)	4.32 (1.4)	-1.1	0.27

Analyses were done using either a Student's t-test or chi-square tests

Supplementary Table 2. **Pearson's correlation results of psychotic-like experiences with functional network size**

Network	[a]ASR Q.40 "hearing"	[a]ASR Q.70 "seeing"	[a]ASR Q.84 "do things"	[a]ASR Q.85 "thoughts"
**1. Default Mode**				
r	**-0.083**	-0.038	-0.046	-0.005
p	**0.01***	0.25	0.16	0.88
**2. Visual**				
r	-0.042	**-0.073**	-0.025	0.003
p	0.20	**0.025***	0.44	0.93
**3. Frontoparietal**				
r	0.003	0.030	0.031	-0.016
p	0.92	0.36	0.34	0.63
**4. Dorsal Attention**				
r	0.003	0.016	0.052	0.020
p	0.93	0.61	0.11	0.55
**5. Language**				
r	**0.115**	**0.082**	**0.066**	0.020
p	**0.0004*****	**0.012***	**0.045***	0.55
**6. Cingulo-Opercular**				
r	**-0.079**	-0.062	**-0.089**	**-0.093**
p	**0.015***	0.058	**0.006***	**0.004****
**7. Sensorimotor (Body)**				
r	0.040	0.052	0.004	0.025
p	0.22	0.11	0.91	0.45
**8. Sensorimotor (Face)**				
r	-0.056	-0.014	0.054	0.031
p	0.086	0.67	0.098	0.34

Bold and *<0.05. Bold and **<0.005. Bold and **<0.0005.
[a]Question items from the Auerbach Adult Self-Report Scale. **Q.40, "hearing": "**I hear sounds or voices that other people think aren't there". **Q.70, "seeing": "**I see things that other people think aren't there". **Q.84, "do things": "**I do things that other people think are strange". **Q.85, "thoughts": "**I have thoughts that other people would think are strange".

<u>Supplementary Table 3.</u> Group differences in functional network brain surface area in bipolar disorder, schizophrenia and HCP-YA participants without a history of psychotic experiences.

Network	HCP-YA (n=712)	BPD (n=35)	SCZ (n=27)	F[a]	p
**1. Default Mode**	0.183 (0.03)	0.175 (0.03)	0.176 (0.03)	2.2	0.11
**2. Visual**	0.162 (0.02)	0.153 (0.02)*	0.153 (0.02)*	**5.1**	**0.006**
**3. Fronto-Parietal**	0.123 (0.02)	0.120 (0.02)	0.114 (0.03)	3.3	0.04
**4. Dorsal Attention**	0.099 (0.02)	0.108 (0.02)*	0.115 (0.03)*	**11.0**	**<0.0001**
**5. Language**	0.073 (0.02)	0.079 (0.02)	0.091 (0.03)*	**8.3**	**0.0003**
**6. Cingulo-Opercular**	0.083 (0.02)	0.081 (0.02)	0.071 (0.02)	4.2	0.02
**7. Sensorimotor (Body)**	0.075 (0.02)	0.075 (0.02)	0.081 (0.04)	0.8	0.4
**8. Sensorimotor (Face)**	0.044 (0.02)	0.0375 (0.01)	0.040 (0.01)	3.6	0.03

[a]Type III sum of squares used in ANOVA
Statistically significant (p<0.0063) results are bolded. Groups showing significant results against HCP-YA is asterisked.

Supplementary Table 4. **Pearson's correlation results of clinical scores against functional network area in Connectom-SCZ subjects.**

Network	SAPS positive symptoms	SAPS disorganized symptoms	SANS	WERCAP affectivity	WERCAP psychosis
**1. Default Mode**					
r	0.017	0.004	0.048	0.162	0.374
p	0.93	0.98	0.81	0.42	0.054*
**2. Visual**					
r	0.146	0.226	0.142	-0.129	-0.231
p	0.47	0.26	0.48	0.52	0.25
**3. Frontoparietal**					
r	-0.139	0.012	-0.029	-0.220	-0.030
p	0.49	0.95	0.89	0.27	0.88
**4. Dorsal Attention**					
r	0.096	-0.043	0.137	**0.502**	0.338
p	0.63	0.83	0.50	**0.008****	0.08*
**5. Language**					
r	-0.006	-0.250	-0.060	-0.231	-0.378
p	0.98	0.21	0.76	0.25	0.059*
**6. Cingulo-Opercular**					
r	-0.206	0.020	0.220	0.167	0.304
p	0.30	0.92	0.91	0.40	0.12
**7. Sensorimotor (Body)**					
r	0.193	0.145	-0.040	-0.190	-0.309
p	0.33	0.47	0.84	0.34	0.12
**8. Sensorimotor (Face)**					
r	0.137	-0.060	-0.018	0.190	0.122
p	0.50	0.76	0.93	0.34	0.55

*<0.1. Bold and **<0.05.

Supplementary Figure 1:

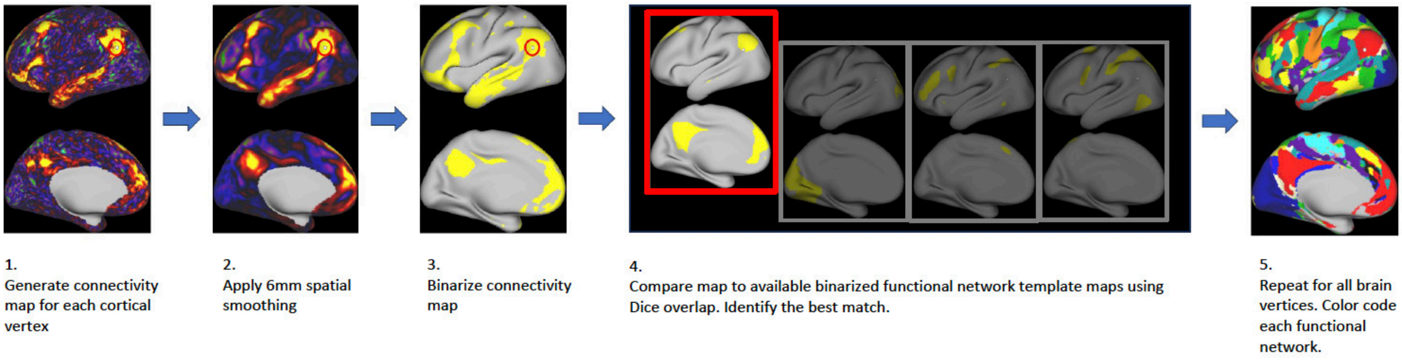

Supplementary Figure 2:

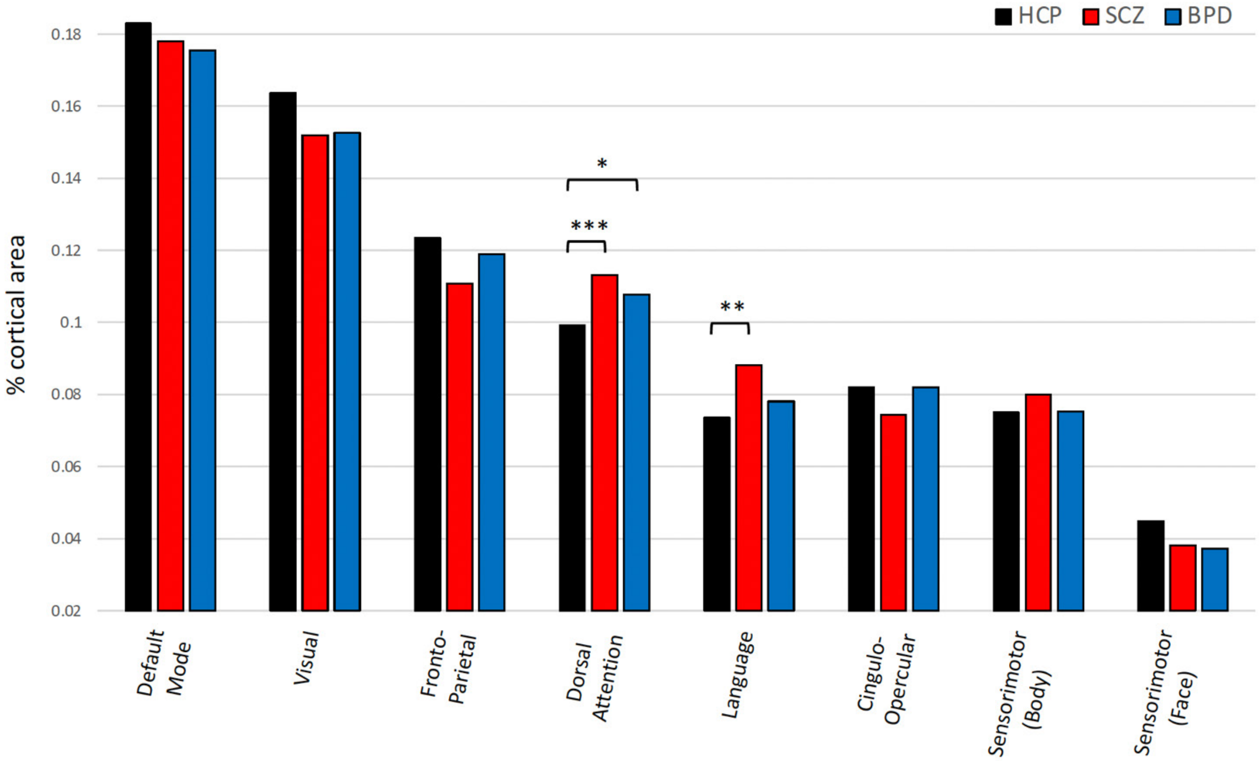

Supplementary Figure 3:

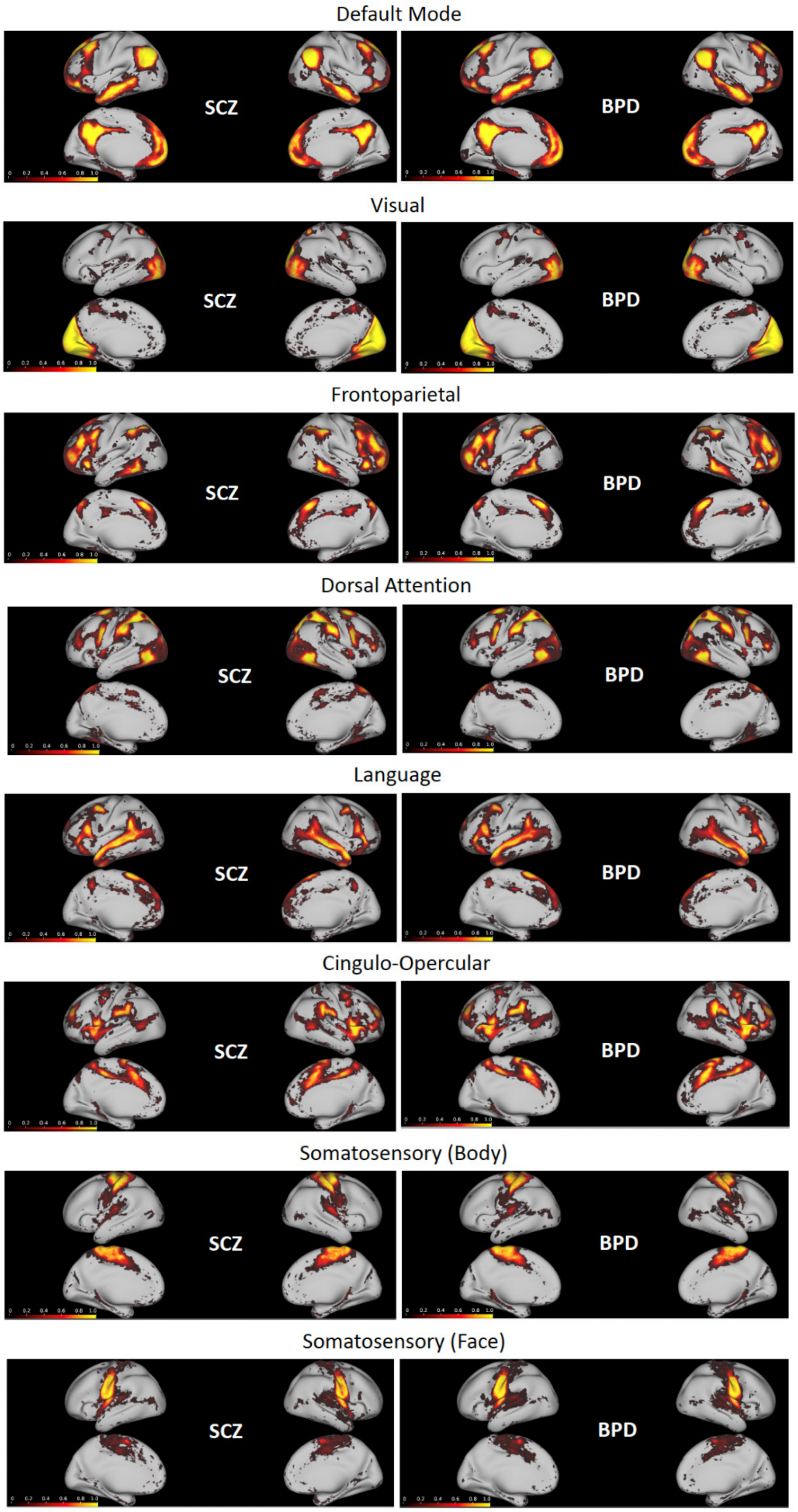

Supplementary Figure 4:

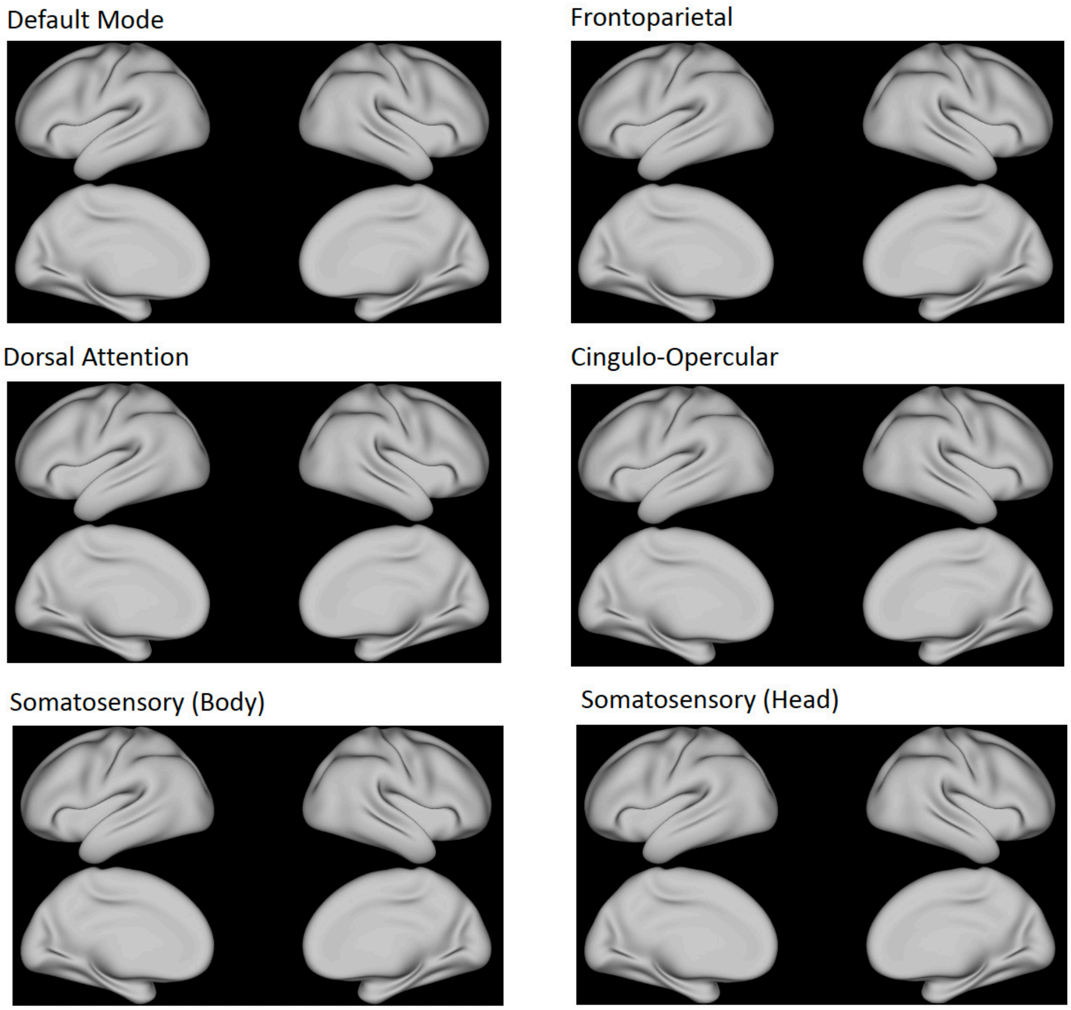

Supplementary Figure 5:

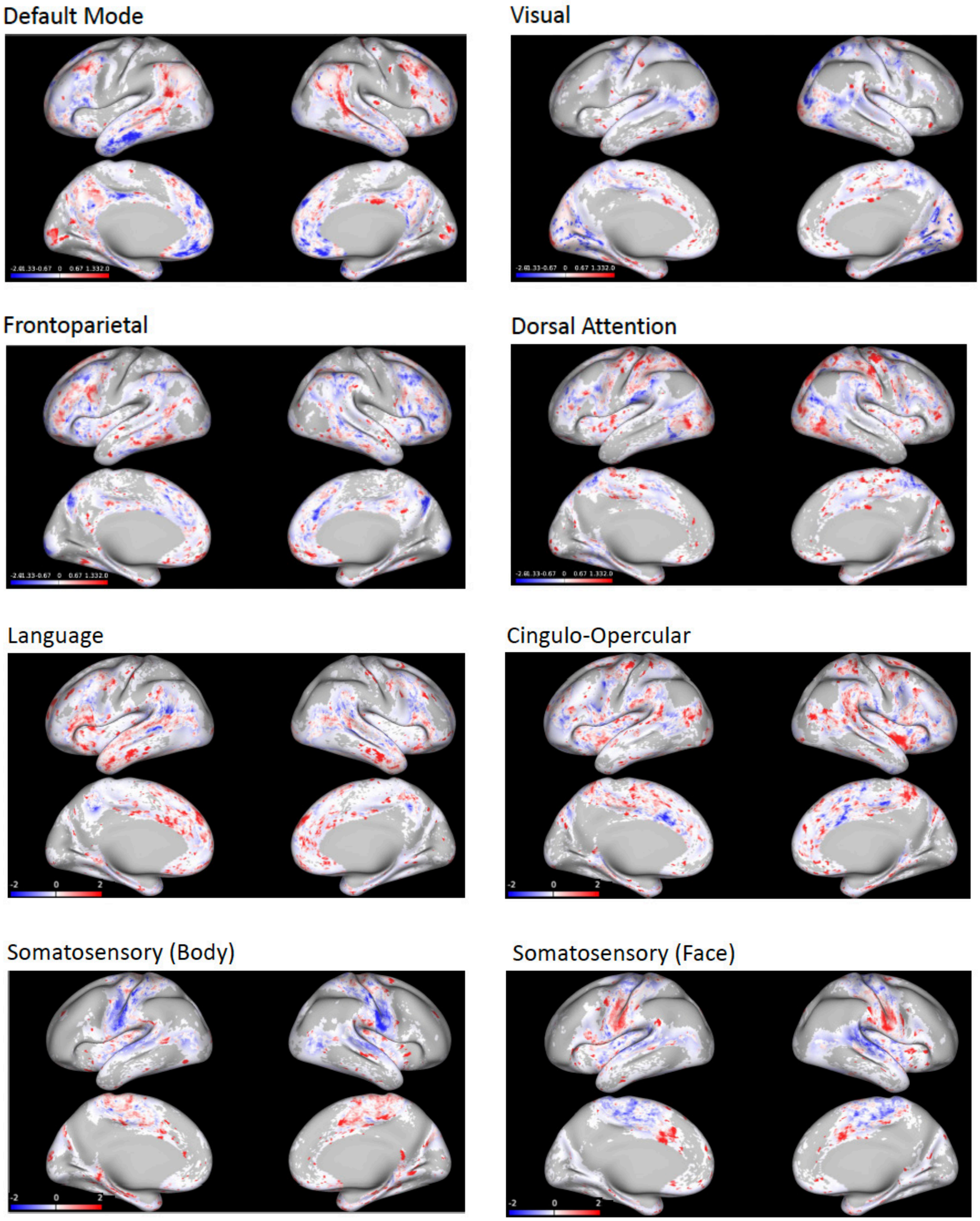

SUPPLEMENTARY FIGURES:

Supplementary Figure 1: Template matching of the brain cortex to identify functional networks. For every participant, a functional connectivity map of each point (vertex) on the cortical surface is generated. This map is then binarized based on the top 5% of values. The binarized map is compared to 12 functional network template maps using Dice coefficient of overlap. The cortical point is assigned the functional network identity of the template with which it has the highest overlap. The matching process is repeated for each cortical point across the brain. The 12 functional networks are distinguished by color coding.

Supplementary Figure 2: Size of functional networks in schizophrenia and bipolar disorder, after correcting for potential confounding effects. The figure compares the least square means cortical surface areas of eight functional networks (relative to the total cortical area) across schizophrenia (n=27) and bipolar disorder (n=35) participants and the healthy control, Human Connectome Project – Young (HCP-YA) cohort (n=1,003), after correcting for sex, age and signal-to-noise ratio. *p<0.01; **p<0.001; ***p<0.0001.

Supplementary Figure 3: Variability in functional network topography across schizophrenia and bipolar disorder participants. Figures show probability maps of the cortical surface distribution of the eight functional networks across schizophrenia (SCZ) and bipolar disorder (BPD) participants. Bright yellow indicates regions where the network is located in close to 100% of participants. Black/dark red regions indicate a lower probability of network localization.

<u>Supplementary Figure 4:</u> Functional network topography in schizophrenia with cluster-correction. The figures are the results of chi-square tests at each brain voxel, comparing the rate of the network localizing to that voxel in schizophrenia participants (n=27) to that voxel in HCP-YA (healthy control) participants (n=1,003) after cluster-based correction. Red shading indicates voxels where the network localizes more frequently in schizophrenia participants. Blue shading indicates voxels where the network localizes more frequently in HCP-YA participants.

<u>Supplementary Figure 5:</u> Functional network topography in bipolar disorder. The figures are the results of chi-square tests at each brain voxel, comparing the rate of the network localizing to that voxel in bipolar disorder participants (n=35) to that voxel in HCP-YA (healthy control) participants (n=1,003), after cluster-based correction. Red shading indicates voxels where the network localizes more frequently in schizophrenia participants. Blue shading indicates voxels where the network localizes more frequently in HCP-YA participants.
